# Comprehensive molecular analysis to predict the efficacy of chemotherapy containing bevacizumab in patients with metastatic colorectal cancer

**DOI:** 10.32604/or.2023.030374

**Published:** 2023-09-15

**Authors:** SUNG HEE LIM, HEE JIN CHO, KYOUNG-MEE KIM, HO YEONG LIM, WON KI KANG, JEEYUN LEE, YOUNG SUK PARK, HEE CHEOL KIM, SEUNG TAE KIM

**Affiliations:** 1 Division of Hematology/Oncology, Department of Medicine, Samsung Medical Center, Sungkyunkwan University School of Medicine, Gangnam-gu, Seoul, 06351, Korea; 2 Precision Medicine Research Institute, Samsung Medical Center, Gangnam-gu, Seoul, 06351, Korea; 3 Department of Biomedical Convergence Science and Technology, Kyungpook National University, Daegu, 41566, Korea; 4 Department of Pathology and Translational Genomics, Samsung Medical Center, Sungkyunkwan University School of Medicine, Seoul, 06351, Korea; 5 Department of Surgery, Samsung Medical Center, Sungkyunkwan University School of Medicine, Gangnam-gu, Seoul, 06351, Korea

**Keywords:** Bevacizumab, Whole-exome sequencing, Ribonucleic acid sequencing, Methylation array, Colorectal cancer

## Abstract

**Background:**

Although bevacizumab is an important treatment for metastatic colorectal cancer (CRC), not all patients with CRC benefit from it; in unselected patient populations, only modest survival benefits have been reported.

**Methods:**

We evaluated clinical outcomes in 110 patients using comprehensive molecular characterization to identify biomarkers for a response to bevacizumab-containing treatment. The molecular analysis comprised whole-exome sequencing, ribonucleic acid sequencing, and a methylation array on patient tissues.

**Results:**

Genomic and molecular characterization was successfully conducted in 103 patients. Six of 103 CRC samples were hypermutated, and none of the non-hypermutant tumors were microsatellite unstable. Among those 103 patients, 89 had adenocarcinoma (ADC), 15 were diagnosed with mucinous ADC, and six had signet-ring cell carcinoma (SRCC). Consensus molecular subtype (CMS) 2 was unique to ADC. Of the four SRCCs, two were CMS1, one was CMS4, and the other was CMS3. *APC* mutation status was a significantly enriched factor in responders to bevacizumab treatment. Fibroblast growth factor receptor (FGFR) 1/2 signaling was upregulated in non-responders, whereas cell cycle, transfer ribonucleic acid processing, nucleotide excision repair, and oxidative phosphorylation pathways were enriched in responders. In addition, *IGF1* was differentially expressed in non-responders (log2 fold change = −1.43, *p* = 4.11 × 10^−5^, false discovery rate = 0.098), and *FLT1* was highly methylated in non-responders (*p* = 7.55 × 10^−3^). When the molecular pathways were reanalyzed separately according to the backbone chemotherapy (FOLFOX *vs*. FOLFIRI), the significance of the molecular pathways varied according to the backbone chemotherapy.

**Conclusions:**

This study sought a subset of CRC patients with a distinct clinical response to chemotherapy containing bevacizumab. Our results need to be validated in a large group of homogenous patient cohort and examined according to the different chemotherapy backbones to create personalized therapeutic opportunities in CRC.

## Introduction

Colorectal cancer (CRC) accounts for 8.5% of all tumor-related mortality and is the fourth most common cause of cancer death [1]. CRC resection offers a good prognosis, but 20%–25% of patients already have metastatic lesions at their initial diagnosis, and approximately half of patients who have a CRC resection eventually experience advanced disease [2]. The prognosis for patients with metastatic CRC is extremely poor. Several factors contribute to improved clinical outcomes [3], and the introduction of novel therapies molecularly targeting either epidermal growth factor signaling, or angiogenesis has been a key development [4–7].

Vascular endothelial growth factors (VEGFs), particularly VEGF-A, have been identified as key factors inducing tumor angiogenesis [8–10]. VEGFs can stimulate the proliferation and survival of endothelial cells and increase the permeability of vessels, thereby supporting the metabolic demands of growing tumors [11]. Treatment with bevacizumab, a monoclonal antibody directed against VEGF, could decrease present tumor vasculature and prevent the growth of new blood vessels, thereby impeding tumor progress. Bevacizumab-based therapy has improved the survival of patients with metastatic CRC [4,5,12,13]. The Food and Drug Administration approved bevacizumab as a first- or second-line treatment joined with chemotherapy for metastatic CRC, based on the survival benefit shown in a landmark trial.

Although bevacizumab has played an important role in treating metastatic CRC, not all patients with CRC benefit from it; in unselected patient populations, only modest survival benefits have been reported [14,15]. Identifying novel biomarkers that could determine which patients are likely to benefit from bevacizumab is thus essential because CRC is a heterogeneous disease consisting of several subtypes with distinct molecular traits [16,17]. In this study, we directly compare clinical outcomes with results from a comprehensive molecular characterization to identify novel biomarkers that could maximize the effective use of bevacizumab in patients with CRC.

## Materials and Methods

### Patient enrollment

Among the patients with metastatic CRC who received bevacizumab-containing chemotherapy as a first- or second-line treatment at Samsung Medical Center between January 2018 and January 2019, 110 patients with samples available for a comprehensive molecular analysis were included in this study. The molecular analysis comprised whole-exome sequencing, ribonucleic acid (RNA) sequencing, and a methylation array of tumor and matched normal patient tissues. Data from the medical records of each patient (age, sex, primary tumor site, histologic type, range of metastasis, treatments, and treatment outcomes) were also examined, particularly the outcomes of bevacizumab treatment. This study was approved by the Institutional Review Board of Samsung Medical Center (#2019-08-064) and was conducted in agreement with the ethical principles of the Declaration of Helsinki and Korean Good Clinical Practice guidelines. All patients provided written informed consent, which included the disclosure of information about the purpose, benefit, and potential risks of this study, the competency of patients to make a decision, and the voluntary nature of the decision to participate.

### Tumor sample collection

Tumor tissues were obtained prior to the start of bevacizumab treatment. If the tumor content was ≥40% after pathological assessment, tumor deoxyribonucleic acid (DNA) and RNA were mined from freshly acquired tissues using a QIAamp mini kit (Qiagen, Hilden, Germany) in accordance with the manufacturer's instructions. For DNA, we used RNaseA (cat. #19101; Qiagen). We determined the concentrations and absorbance ratios (OD_260_/OD_280_ and OD_260_/OD_230_) with an ND1000 spectrophotometer (NanoDrop Technologies, Thermo Fisher Scientific, MA, USA) and quantified DNA/RNA using a Qubit fluorometer (Life Technologies, CA, USA).

### Whole-exome sequencing for tumor tissue

To generate standard exome capture libraries, we used the Agilent SureSelect Target Enrichment protocol for an Illumina paired-end sequencing library (ver. B.3, June 2015) with 200 ng of input formalin-fixed, paraffin-embedded DNA. In all cases, the SureSelect Human All Exon V5 probe set was used. DNA quantity and quality were evaluated using PicoGreen [18] and NanoDrop [19]. Fragmentation of 1 μg of genomic DNA was performed using adaptive focused acoustic technology (Covaris Inc., MA, USA). The fragmented DNA was repaired, an “A” was ligated to the 3 end, and Agilent adapters were ligated to the fragments. Once ligation had been assessed, the adapter-ligated product was amplified using polymerase chain reaction (PCR). Subsequently, the final purified product was quantified using quantitative PCR (qPCR), as directed in the qPCR Quantification Protocol Guide, and assessed using a Caliper High Sensitivity DNA LabChip kit (PerkinElmer Inc., MA, USA). For exome capture, 250 ng of the DNA library was mixed with hybridization buffers, blocking mixes, RNase block, and 5 µl of the SureSelect all exon capture library, according to the standard Agilent SureSelect Target Enrichment protocol. Hybridization to capture baits was performed at 65°C using the heated lid option on a PCR machine with a thermocycler at 105°C for 24 h. The captured DNA was amplified. The final purified product was quantified using qPCR according to the qPCR Quantification Protocol Guide and assessed using a TapeStation RNA ScreenTape (Agilent). Finally, we performed sequencing using the HiSeq™ 2500 platform (Illumina, San Diego, USA).

### Whole exome sequencing data analysis

The reads resulting from the whole exome sequencing (WES) were mapped to human genome version 19 (hg19) using the Burrows-Wheeler Aligner (version 0.7.12-r1039) and the BWA-MEM algorithm [20]. SAMtools sorted the aligned sequences by genomic coordinates (v0.1.19) [21]. The sorted reads were subjected to Genome Analysis Toolkit (GATK) v3.6 and v4.13 for duplicate marking, indel realignment, and base recalibration [22]. We performed MuTect2 from GATK v4.13 to detect tumor somatic mutations in the recalibrated bam files from the tumor and matched normal samples. After removing possible germline events (population allele fraction <2.5e-6 in gnomAD), we annotated the called mutations using variant effect predictors [23]. The resulting variant events were converted to maf format, and then the variants with <4 altered reads were eliminated from further analysis in R. The COSMIC v3 single base substitution mutational signatures of each tumor sample were determined using deconstructSigs (v1.8.0, R package) [24,25]. The ngCGH Python package was used to estimate somatic copy number variations in tumor samples by comparison with the matched normal samples.

### RNA sequencing

The total RNA concentration was estimated using Quant-IT RiboGreen (Invitrogen). To determine the DV200 value (% of RNA fragments >200 bp), samples were run on the TapeStation RNA ScreenTape (Agilent). Overall, 100 ng of total RNA was subjected to sequencing library construction using a TruSeq RNA Access Library prep kit (Illumina, San Diego, CA, USA) according to the manufacturer’s protocol. Briefly, total RNA was first fragmented into small pieces using divalent cations at an elevated temperature. The cleaved RNA fragments were copied into first-strand complementary DNA (cDNA) using SuperScript II reverse transcriptase (Invitrogen, #18064014) and random primers. That was followed by second-strand cDNA synthesis using DNA polymerase I, RNase H, and deoxyuridine 5-triphosphate. The cDNA fragments were subjected to an end-repair process, the addition of a single “A” base, and ligation of the adapters. Thereafter, the products were purified and enriched with PCR to create a cDNA library. All libraries were normalized, and six were pooled into a single hybridization/capture reaction. The pooled libraries were incubated with a cocktail of biotinylated oligos corresponding to the coding regions of the genome. Targeted library molecules were captured with hybridized biotinylated oligo probes using streptavidin-conjugated beads. After two rounds of hybridization/capture reactions, the enriched library molecules were subjected to a second round of PCR amplification. The captured libraries were quantified using a KAPA Library Quantification kit for the Illumina Sequencing platforms according to the qPCR Quantification Protocol Guide (KAPA BIOSYSTEMS, #KK4854) and assessed using the TapeStation D1000 ScreenTape (Agilent Technologies, #5067-5582). The indexed libraries were subsequently submitted to the Illumina HiSeq 2500 platform (Illumina, Inc., San Diego, CA, USA), and paired-end (2 × 100 bp) sequencing was performed by Macrogen Inc., Seoul, Korea.

### Gene expression calling

RNA sequence reads were mapped on hg19 by STAR (v2.6.1d) [26] and sorted according to the genomic coordinates. Cufflinks (v2.2.1) was used to calculate gene expression levels in fragments per kilobase million (FPKM) [27] using Ensembl gene annotation. The FPKM values were log2-transformed for further analysis. CMSclassifier (R package) and ESTIMATE (R package) determined the consensus molecular subtype (CMS) of the tumor tissues and tumor purity, respectively [17,28]. The expression scores of gene sets were estimated using the single sample gene set enrichment analysis (ssGSEA) algorithm in GSVA (R package) [29]. The gene set enrichment analysis (GSEA) was performed on GSEA-P [30], and the networks based on the GSEA results were generated using Cytoscape (v3.7.1) Enrichment Map [31]. To identify differentially expressed genes (DEGs) between responders and non-responders to bevacizumab, the DEseq2 (R package) was used [32].

### Methylation profiling array

DNA from 75 CRC tumor samples was subjected to the Infinium MethylationEPIC Array to acquire genome-wide DNA methylation profiles in CRC tissues. The raw idat files were processed to generate normalized β-values for each probe using the minfi R package [33]. We selected the top 1500 most variable probes (based on standard deviation) from the normalized β-values and applied an unsupervised hierarchical clustering analysis to the filtered normalized data. Then, we sorted the CpG island methylator phenotypes (CIMPs) into CIMP-high, CIMP-low, and CIMP-negative according to the clusters. To summarize the β-values in each gene level, we first eliminated the probes that (1) were located in chromosome X or Y, (2) had a single nucleotide polymorphism (SNP)-associated distance <10 bp, or (3) were not located in a promoter region (transcription start site >1500 bp). Then, for each sample, the mean β-value of the promoter regions was calculated to represent the promoter methylation level for each gene. In that way, we obtained the promoter methylation levels for 18,226 genes from 75 tumor samples. To compare the gene promoter methylation levels of responders and non-responders, student’s *t*-test was performed for each gene.

### Statistical analysis

Patient characteristics were summarized using descriptive statistics. The response rate (RR) was calculated as the percentage of patients who experienced a confirmed complete response (CR) or partial response (PR), and the disease control rate (DCR) was calculated as RR + stable disease (SD), following the RECIST 1.1 guidelines. Each nominal variable was compared using Fisher’s exact test or the χ2 test. Survival outcomes were estimated using the Kaplan-Meier method and compared using the log-rank test. The associations between genetic alterations and responses to bevacizumab-containing treatment were analyzed using R; other analyses were conducted using the Statistical Package for the Social Sciences version 19.0 (SPSS Inc., Chicago, IL, USA).

## Results

### Patient characteristics

Baseline patient characteristics are presented in Table 1. The median age was 61 years (range: 35–79), and the numbers of females and males were 62 (56%) and 48 (44%), respectively. The most common pathologic differentiation was the moderate type (n = 67%, 61%), and the primary tumor locations were the left side (58%) and right side (42%). *BRAF*-mutated tumors were found in 11 patients (10%), and the most common *BRAF* mutation was V600E (N = 8), with L597Q, D594G, and E83G mutations identified in one patient each. The median Eastern Cooperative Oncology Group performance status score was 1. Most patients (83%) received bevacizumab treatment as the first-line treatment, and 60% of the patients received treatment with fluorouracil, irinotecan, and leucovorin as the backbone chemotherapy and 40% of the patients were treated with fluorouracil, oxaliplatin and leucovorin as the backbone chemotherapy.

**Table 1:** Patients’ characteristics (N = 110)

		N (%)
Age	61 (35–79)	
Gender	Male	48 (44%)
	Female	62 (56%)
Pathologic differentiation	Poorly	15 (14%)
	Moderate	67 (61%)
	Well	7 (6%)
	Mucinous	15 (14%)
	Signet ring cell	6 (5%)
Primary tumor	Right	46 (42%)
	Left	64 (58%)
Mutational status of KRAS	Mutation	47 (43%)
	Wild type	56 (51%)
	N.E.	7 (6%)
Status of microsatellite instability (by IHC)	MSS	80 (73%)
	MSI	5 (4%)
	N.E.	25 (23%)
Mutational status of BRAF	Mutation	11 (10%)
	Wild type	99 (90%)
ECOG performance status	0	27 (25%)
	1	83 (75%)
Line of bevacizumab containing chemotherapy	First	91 (83%)
	Second	19 (17%)
Chemotherapy backbone with bevacizumab	FOLFOX	44 (40%)
	FOLFIRI	66 (60%)

Note: MSS, microsatellite stable; MSI, microsatellite instability; FOLFOX, fluorouracil; leucovorin, and oxaliplatin; FOLFIRI, fluorouracil, leucovorin, and irinotecan; IHC, immunohistochemistry; ECOG, Eastern Cooperative Oncology Group; N.E., not evaluable.

Of the 110 patients, 37 showed a PR (33%), 57 achieved SD (52%), and 16 had progressive disease (PD) (15%) (Table 2). The overall confirmed RR and DCR were 33% and 85%, respectively.

**Table 2:** The efficacy of bevacizumab containing therapy

	Bevacizumab containing treatment		
	1^st^ line (N = 91)	2^nd^ line (N = 19)	Overall (N = 110)
CR	–	–	
PR	32	5	37
SD	45	12	57
PD	14	2	16
Responder	32	5	37
Response rate	35.2%	26.3%	33.6%

Note: CR, complete response; PR, partial response; SD, stable disease; PD, progressive disease.

No significant difference in the patient characteristics between the response group and non-response groups (Suppl. Table S1).

### Genomic landscape and correlates of response to bevacizumab

**Figure 1:**
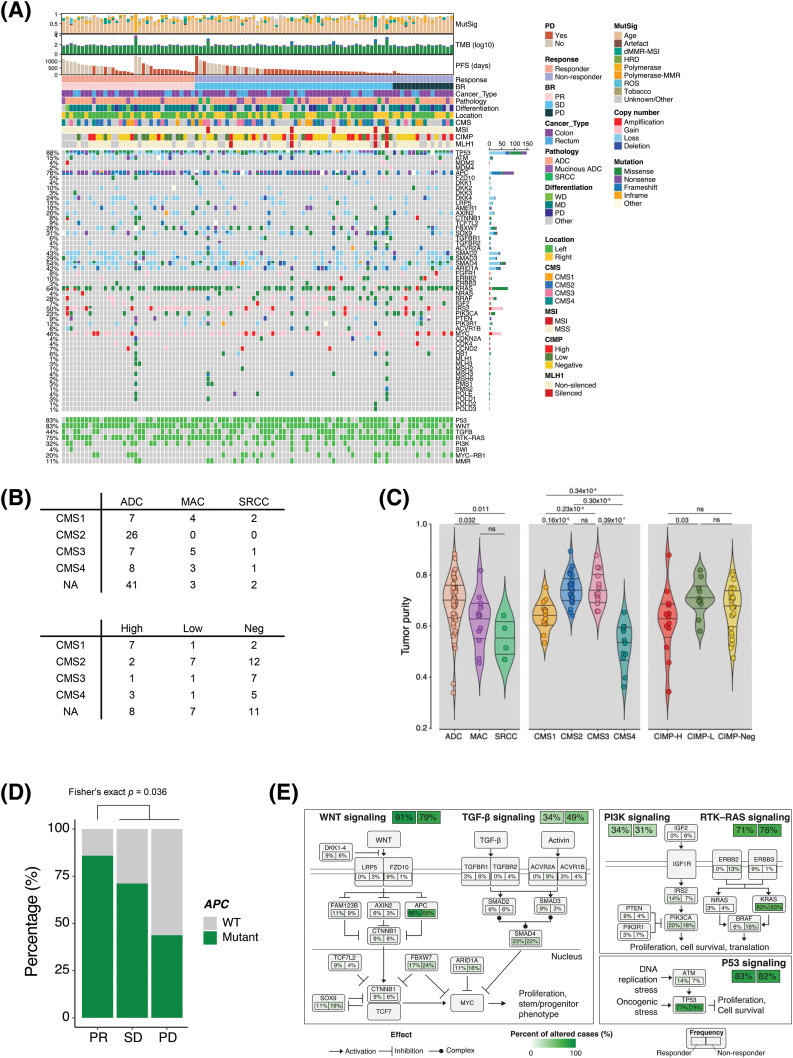
Genomic landscapes and correlates of the response to bevacizumab. (A) Genomic landscape of 103 patients with CRC who received bevacizumab. (B) CMS classification of patients with CRC according to pathology (top) and CIMP status (bottom). (C) Violin plot of the ESTIMATE tumor purity in CRC patients based on pathology (left), CMS classification (middle), and CIMP status (right). Wilcoxon rank-sum *p* values are shown. (D) Bar plot of the frequency of *APC* mutation in responders (CR and PR) and non-responders (SD and PD) to bevacizumab treatment. Fisher’s exact *p* value is shown. (E) Summary of CRC core pathway alteration frequency. CR, complete response; PR, partial response; SD, stable disease; PD, progressive disease. CMS, consensus molecular subtype; NA, not available; ADC, adenocarcinoma; MAC, mucinous adenocarcinoma; SRCC, signet-ring cell carcinoma; NEG, negative; WT, wild type; CIMP, CpG island methylator phenotype.

To characterize the genomic and molecular features that dictate the response to bevacizumab, we performed whole-exome sequencing, RNA sequencing, and a methylation array on tumor and matched normal tissues derived from 103 patients with CRC (Fig. 1A). Seven of the 110 patients had insufficient samples for the analysis. Of the 103 CRC samples, six were hypermutated, five were microsatellite instable (MSI), and three showed *MLH1* epigenetic silencing (Fig. 1A). None of the non-hypermutant tumors was MSI. The ultra-hypermutant tumor (#40) was microsatellite stable; however, it was *POLE*-mutant and accordingly displayed a high proportion of *POLE*-induced mutational signatures (Suppl. Fig. S1A). MutSigCV identified the significantly mutated genes in the CRC cohort as follows: adenomatous polyposis coli (*APC*, 72%), *SOX9* (17%), *TP53* (77%), *KRAS* (61%), *SMAD4* (19%), and *FBWX7* (21%), which is similar to the results of previous studies (Suppl. Fig. S1B) [16]. Among the 110 CRC patients, 89 were diagnosed with adenocarcinoma (ADC), 15 were diagnosed with mucinous ADC, and the other 6 were diagnosed with signet-ring cell carcinoma (SRCC). The three different pathologies displayed distinct molecular traits. The CMS2 subtype, which is a canonical subtype characterized by low stromal/immune infiltration [17], was unique to ADC (Fig. 1B). Consistently, ADC showed significantly higher tumor purity than the other CRC types (Fig. 1C). Moreover, tumor microenvironment infiltration was high in SRCC, and tumor purity was low (Fig. 1C). Of the four SRCCs, two were CMS1 (immune activation), one was CMS4 (mesenchymal subtype), and the other was CMS3 (metabolic subtype) (Fig. 1D). In addition, we classified the tumor samples as CIMP-high, CIMP-low, and CIMP-negative using unsupervised clustering of DNA methylation (Suppl. Fig. S2A). As expected, the tumor purities were lower in CIMP-high samples than in CIMP-low and CIMP-negative samples, and >50% of the CIMP-high samples were identified as the CMS1 subtype (Fig. 1B).

Subsequently, we examined the molecular and genomic correlates of responses to bevacizumab. CIMP-low tumors occurred significantly more frequently in responders (Fisher’s exact *p* = 0.036, odds ratio [OR] = 3.47); however, the CMS subtype and tumor purity were not associated with bevacizumab sensitivity (Suppl. Figs. S3A and S3B). Among the genomic alterations in the CRC core pathways [16], *APC* mutation status was a significantly enriched factor in responders (Fig. 1D and Suppl. Fig. S3C). *APC* mutation occurred in 72% of all CRC samples and was significantly predominant in responders (30/35 *vs*. 44/68, Fisher’s exact *p* = 0.036, OR = 3.24). Specifically, only 43.8% of patients with CRC who experienced PD after bevacizumab treatment possessed *APC* mutations (Fig. 1D). We summarized the alteration frequencies of CRC core pathways according to the response (Fig. 1E).

### IGF1-FGFR1 pathway activation in bevacizumab-resistant patients

**Figure 2:**
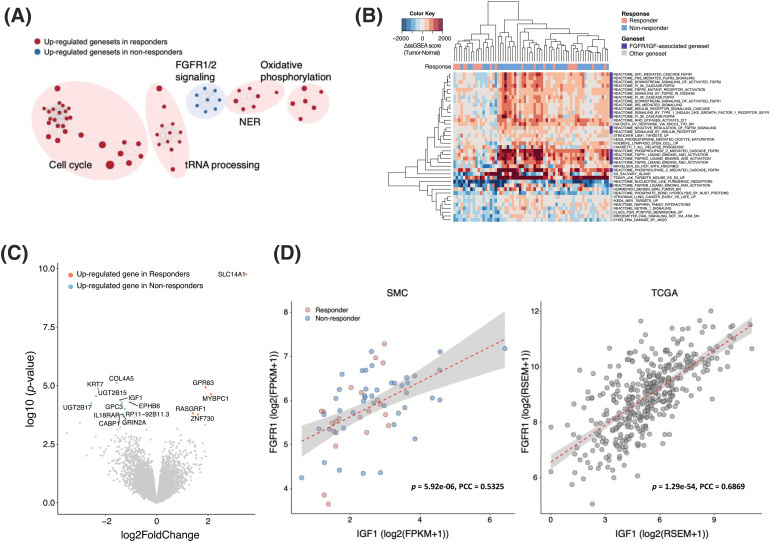
IGF1-FGFR signaling activation in bevacizumab-resistant colorectal cancers. (A) Clustering of significantly enriched pathways in responders (red) and non-responders (blue). (B) Gene sets with significantly higher ΔssGSEA scores in non-responders compared with responders. ΔssGSEA indicates the difference in ssGSEA scores between tumors and matched normal samples (ssGSEA_tumor_-ssGSEA_normal_). (C) Volcano plot of differentially expressed gene analysis (absolute log2 fold changes ≥1 and FDR <0.25) between responders (red) and non-responders (blue). X-axis represents log2-scale fold change; Y-axis, −log10 *p* value. (D) Correlation between the expression levels of *IGF1* (X-axis) and *FGFR1* (Y-axis) in the SMC (left) and TCGA CRC (right) datasets.

Transcriptomic profiles were made using RNA sequencing to reveal the potential mechanism behind resistance to bevacizumab treatment in CRC patients. To identify the molecular pathways enriched in the bevacizumab-resistant group, we applied a GSEA to the gene expression profiles and found that fibroblast growth factor receptor (FGFR) 1/2 signaling was upregulated in non-responders, whereas cell cycle, transfer RNA (tRNA) processing, nucleotide excision repair (NER), and oxidative phosphorylation pathways were enriched in responders (Fig. 2A). In addition, we used the ssGSEA method to calculate differences in the gene set expression levels in tumors and their matched normal tissues (ΔssGSEA scores) and compare the pathways up-/downregulated between them. We also compared the ΔssGSEA scores of the molecular pathways between responders and non-responders. Interestingly, in accordance with previous GSEA results, FGFR and IGF signaling was significantly upregulated in the tumors of non-responders compared with those of responders (Fig. 2B). When we analyzed DEGs between responders and non-responders, we found that *IGF1* was a DEG in non-responders (log2 fold change = −1.43, *p* = 4.11 × 10^−5^, false discovery rate = 0.098) (Fig. 2C). To confirm whether *FGFR* and *IGF1* expression levels are correlated, we performed a correlation analysis between them. *FGFR1* expression levels correlated positively with *IGF1* expression levels in both the SMC (r = 0.53) and TCGA CRC (r = 0.69) cohorts (Fig. 2D).

### FLT1 epigenetic silencing

**Figure 3:**
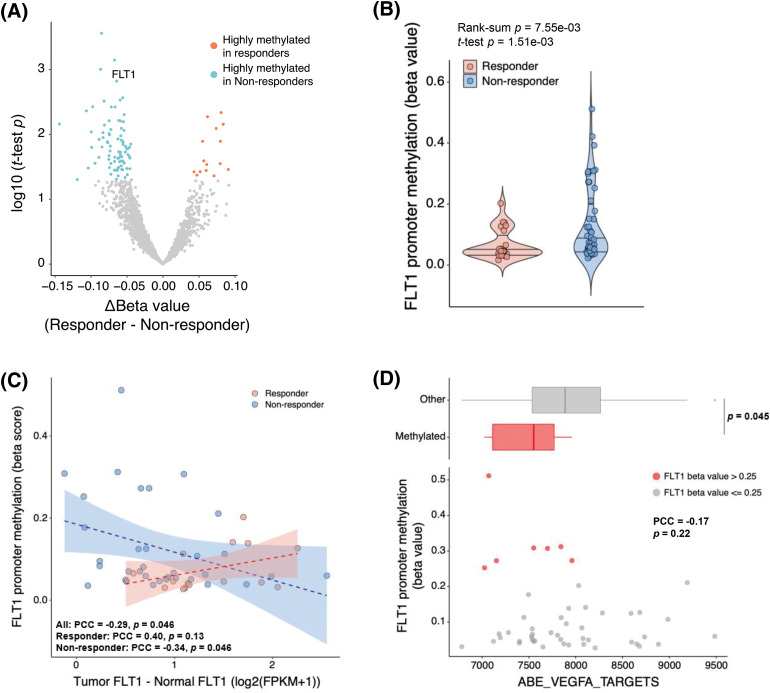
Decreased efficacy of bevacizumab via *FLT1* epigenetic silencing. (A) Volcano plot of results from the differentially methylated gene promoter analysis between responders (red) and non-responders (blue). X-axis represents the difference in each gene β-value between responders and non-responders; Y-axis, −log10 (Student’s *t*-test *p* value). (B) *FLT1* promoter methylation levels in responders and non-responders. (C) Pearson correlation analysis of *FLT1* promoter methylation levels and *FLT1* upregulation levels in tumors compared with matched normal samples (*FLT1* log_2_FPKM_tumor_ − *FLT1* log_2_FPKM_normal_). Blue and red dots indicate non-responders and responders, respectively. Blue and red lines indicate the linear regression line in non-responders and responders, respectively. (D) Correlation between the *FLT1* promoter methylation levels and ssGSEA scores of VEGF-A targets. *FLT1* promoter methylated tumors (β > 0.25) (red dots) showed lower expression of VEGF-A targets than unmethylated samples (upper panel). Wilcoxon rank-sum *p* value is shown.

Because epigenetic regulation is a common resistance mechanism against cancer therapies, we explored DNA methylation profiles to identify epigenomic markers that might predict the bevacizumab response. When the beta values of genes were compared between responders and non-responders, *FLT1* was more highly methylated in non-responders than responders (*p* = 7.55 × 10^−3^) (Figs. 3A and 3B). To examine whether *FLT1* promoter methylation regulates *FLT1* mRNA expression, we examined the difference between *FLT1* expression levels in the tumor and those in matched normal tissue and compared it with *FLT1* promoter methylation levels. We found a negative correlation between *FLT1* promoter methylation levels and the difference in expression levels, especially in non-responders (Fig. 3C). In addition, we confirmed that among non-responders, the expression levels of VEGF-A targets were significantly lower in methylated samples than in *FLT1* promoter unmethylated samples (Fig. 3D).

### Survival analysis

The survival analysis was conducted between the response group and non-response group in the first-line and second-line setting, respectively. Among 91 patients who received bevacizumab in their first-line treatment, the response group showed significantly longer PFS and OS than non-response group. The median PFS and OS were respectively 14.2 months (95% CI 6.6–21.8), 41.7 months (95% CI 10.78–72.56) in the response group compared with 9.4 months (95% CI 7.03–11.83), 28.8 months (95% CI 23.02–34.52) in the non-response group (HR 2.18 95% CI 1.28–3.72, *p* = 0.004 for PFS; HR 1.94 95% CI 1.12–3.36, *p* = 0.018 for OS).

Although not statistically significant in patients (N = 19) treated with bevacizumab as their second-line treatment, the response group showed a better PFS and OS than non-response group. The median PFS and OS were respectively 27.8 months (95% CI 17.82–37.78), 37.9 months (95% CI 27.06–48.68) in the response group compared with 9.0 months (95% CI 3.36–14.64), 12.9 months (95% CI 3.23–22.51) in the non-response group (HR 3.19 95% CI 0.86–11.83, *p* = 0.082 for PFS, HR 2.43 95% CI 0.67-8.85, *p* = 0.179 for OS) (Suppl. Fig. S7).

## Discussion

For this study, we used a comprehensive molecular approach to identify novel biomarkers that might predict the response to bevacizumab in patients with metastatic CRC. Although some previous efforts were made to find potential biomarkers for a response to bevacizumab-containing therapies [34–36], they used insufficient molecular analyses and samples. In this study, all molecular analyses (whole-exome sequencing, RNA sequencing, and a methylation array) were based on tumor and matched normal tissues that were obtained before the initiation of bevacizumab treatment. Thus, our data might be used to select a patient population that could benefit from bevacizumab-based chemotherapy. In this study, *APC* mutation status, FGFR 1/2 signaling pathways, the cell cycle, tRNA processing, NER, oxidative phosphorylation, *IGF1* expression status, and *FLT1* methylation status correlated closely with sensitivity to bevacizumab-containing treatment. Nevertheless, our findings need to be validated in a prospective clinical study of patients with CRC who plan to receive bevacizumab.

Loss of *APC* is the main driver of Wnt signaling in CRC, and the significant role of *APC* has been demonstrated in previous studies [37–39]. Different *APC* mutations result in distinct levels of canonical Wnt pathway activity [40,41]. In this study, *APC* mutation occurred in 72% of all CRC samples and was significantly predominant in responders (30/35 *vs*. 44/68, Fisher’s exact *p* = 0.036, OR = 3.24). Responders also showed a higher alteration frequency in the Wnt signaling pathway than non-responders (91% *vs*. 79%). However, that difference was not statistically significant (32/35 *vs*. 54/68, Fisher’s exact *p* = 0.16). This discrepancy might be attributed to the complexity of the Wnt pathway. The Wnt pathway is commonly classified into β-catenin-dependent (canonical) and β-catenin-independent (non-canonical) signaling [42]. The *APC* gene interacts with the canonical Wnt pathway [37,38]. Additionally, Wnt pathway components, particularly *APC*, have been linked to chromosomal instability (CIN) by multiple mechanisms [43–45]. CIN is frequently observed in CRC and is associated with poor prognosis. According to a previous study, patients with metastatic CRC with high CIN had positive clinical outcomes after bevacizumab combination therapy [46]. However, in our study, the copy number load was not associated with the response to bevacizumab treatment (Suppl. Fig. S3D).

IGF-FGFR signaling controls the growth, migration, survival, and differentiation of numerous cell types [47–49]. This signaling could also indirectly promote angiogenesis. Theoretically, the poor efficacy of anti-VEGF therapies can be attributed to cooperation between this signaling and VEGF-R for angiogenesis. In this study, non-responders showed upregulation of both FGFR1/2 signaling and *IGF1* gene expression. According to publicly available single-cell RNA-seq CRC data, both *IGF1* and FGFR1 are highly expressed in Stromal3 cells (Suppl. Fig. S4), which are a sub-cell group of fibroblasts [50]. Thus, with the help of this subgroup of fibroblasts in the tumor microenvironment, IGF1-FGFR1 signaling activation could be the resistance mechanism against bevacizumab treatment in patients with CRC. Therefore, a therapeutic strategy based on the co-inhibition of FGFR and VEGF-R signaling might be needed for CRC treatment.

In some cancer cells, changes in intracellular VEGF-VEGF-R signaling occur because of the epigenetic silencing of *FLT1* [51]. Cell lines with epigenetic gene silencing of *FLT1* showed insufficient inhibition of proliferation after treatment with VEFG-TKIs [52]. Kim et al. reported that *FLT1* epigenetic silencing is higher in renal cancer tissue from non-responders than responders to anti-angiogenetic agents [53]. In this study, we found that *FLT1* epigenetic silencing via promoter methylation attenuated the therapeutic efficacy of bevacizumab in patients with CRC; a lack of therapeutic target activation was noted. This finding suggests that the promoter methylation status of *FLT1* might be a helpful biomarker for predicting the success of bevacizumab treatment in patients with metastatic CRC.

In this study, we implemented our analysis using a comprehensive molecular approach with tissues to identify novel biomarkers that could predict the response to bevacizumab treatment in patients with metastatic CRC. Previously, much interesting research has examined sensitivity to antiangiogenesis therapy in CRC. Some of those previous studies, unlike our analysis, investigated germline polymorphisms in genes involved in VEGF pathways by using blood samples [54–56]. Given that bevacizumab has a soluble target (VEGF) and not a target on cancer cells, efforts to evaluate biomarkers related to bevacizumab in germline angiogenesis polymorphisms are likely to be meaningful. Currently, no biomarkers are available to select a patient population likely to benefit from bevacizumab. In the future, integrated biomarker-studies that evaluate both tissue and blood might provide more information to guide the use of bevacizumab in CRC patients.

We also separately analyzed the relationship between a response to bevacizumab and various molecular characterizations according to the backbone chemotherapy used (FOLFOX *vs*. FOLFIRI). We found that APC mutation status was not a significantly enriched factor for responders to bevacizumab in both the FOLFOX and FOLFIRI groups (Suppl. Fig. S5A). It was a significant prognostic factor for PFS in the FOLFOX and bevacizumab group (Suppl. Fig. S5B), but not in the FOLFIRI and bevacizumab group. On the other hand, FLT1 was a highly methylated gene in non-responders to bevacizumab treatment in the FOLFIRI and bevacizumab group, but not in the FOLFOX and bevacizumab group (Suppl. Fig. S5C). In other words, when we reanalyzed the molecular pathways separately, their significance depended on the backbone chemotherapy used (FOLFOX *vs*. FOLFIRI) (Suppl. Figs. S6A and S6B).

Our study has several limitations. First, our study population is clinically heterogeneous and thus subject to potential biases. For example, we included 46 CRC patients (42%) with right-sided primary tumors. That high frequency of right-sided primary tumors is unusual in studies of CRC. Also, patients in this study were treated with different backbone chemotherapy regimens alongside bevacizumab. Second, our study included a relatively small number of patients; thus, drawing definite conclusions about molecular biomarkers is difficult. Third, only Asian patients with CRC were analyzed in this study. Differences in genomic profiles and sensitivity to anti-angiogenetic agents exist between Western and Eastern patients with CRC. Fourth, we didn’t analyze the genomic profiles of control samples from patients who received only chemotherapy without bevacizumab. Fifth, we intended to analyze too many variables for our relatively small sample size, which prevented us from adjusting for important factors that might have affected our results.

Generally, the efficacy of adding bevacizumab to chemotherapy is modest. In such cases, the effect of bevacizumab might be reflected in survival times, rather than the tumor response. However, our comprehensive molecular analysis considered many variables, and our study population contained only 110 patients. Thus, it was impossible to conduct a comprehensive genomic analysis for survival. Although we could not analyze the direct associations between various molecular alterations and survival, patients in the response group showed significantly longer PFS and OS than those in the non-response group (Suppl. Fig. S7).

Therefore, our findings must be interpreted with caution, and validation with larger patient samples is warranted.

## Conclusion

We identified multiple molecular and transcriptional features correlated with the clinical response to bevacizumab therapy in patients with CRC. When the molecular pathway data were reanalyzed separately according to the backbone chemotherapy (FOLFOX *vs*. FOLFIRI), the significance of the results differed between groups. We have tried to identify a subset of CRC patients with a distinct clinical response to chemotherapy containing bevacizumab, and our results need to be validated with larger patient samples and different chemotherapy backbones to enable personalized therapeutic opportunities in CRC.

## Supplementary Materials

FIGURE S1 (A) High proportion of *POLE*-induced mutational signatures in the ultra-hypermutant tumor (#40) with microsatellite stable (B) MutSigCV identified the significantly mutated genes in the CRC cohort.

FIGURE S2 Classification the tumor samples as CIMP-high, CIMP-low, and CIMP-negative using unsupervised clustering of DNA methylation.

FIGURE S3 (A) No significant association between the CMS subtype and the bevacizumab sensitivity. (B) No significant association between the tumor purity and the bevacizumab sensitivity. (C) *APC* mutation status was a significantly enriched factor in responders among the genomic alterations in the CRC core pathways. (D) No significant association between the copy number load and the response to bevacizumab treatment.

FIGURE S4 Both *IGF1* and *FGFR1* are highly expressed in Stromal3 cells according to publicly available single-cell RNA-seq CRC data.

FIGURE S5 (A) *APC* mutation status was not a significantly enriched factor for responders to bevacizumab in both the FOLFOX and FOLFIRI groups. (B) *APC* mutation was a significant prognostic factor for PFS in the FOLFOX and bevacizumab group. (C) *FLT1* was a highly methylated gene in non-responders to bevacizumab treatment in the FOLFIRI and bevacizumab group, but not in the FOLFOX and bevacizumab group.

FIGURE S6 (A) Gene Set Enrichment Analysis of the molecular pathways showed *FGFR1* significance depended on the backbone chemotherapy used (FOLFOX *vs.* FOLFIRI) (B) Different IGF expression between responder and non-responder according to the backbone chemotherapy (FOLFIRI *vs.* FOLFOX).

FIGURE S7 Survival analysis of PFS and OS between the patients in the response group and non-response group according to the line of chemotherapy plus bevacizumab.

## Data Availability

All data needed to evaluate the conclusions in the paper are present in the paper and/or the Supplementary Materials. Additional data related to this paper may be requested from the authors.

## Supplementary Materials

The supplemental material for this article is available online at https://doi.org/10.32604/or.2023.030374.
