# Supplementary figures and images for: Comprehensive molecular analysis to predict the efficacy of chemotherapy containing bevacizumab in patients with metastatic colorectal cancer
Source: Oncol Res. 2023 Sep 15;31(6):855–66. doi: 10.32604/or.2023.030374 (PMC10513961; doi:10.32604/or.2023.030374)

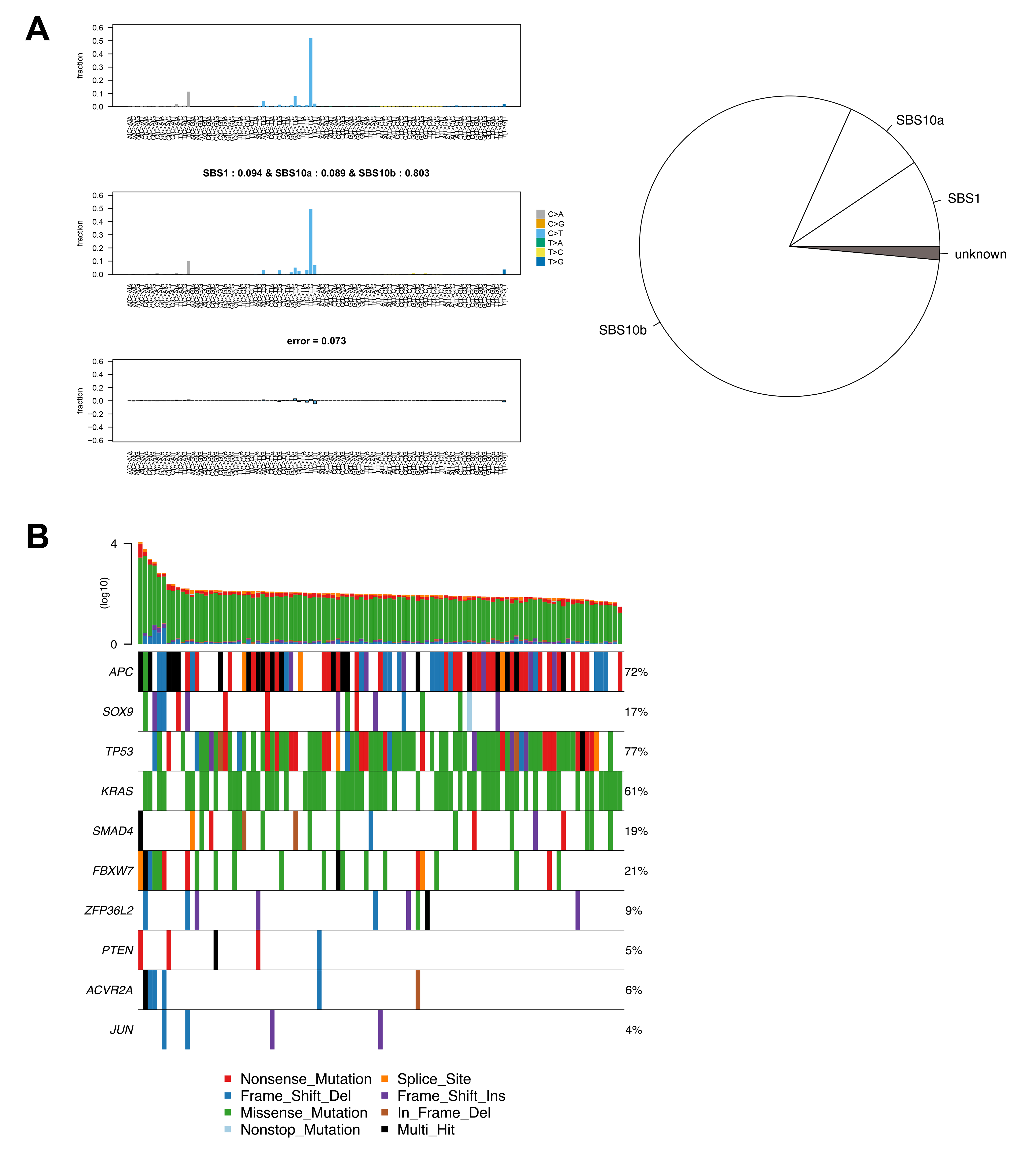

Supplement: FIGURE S1 [file OncolRes-31-30374-s001.tif]

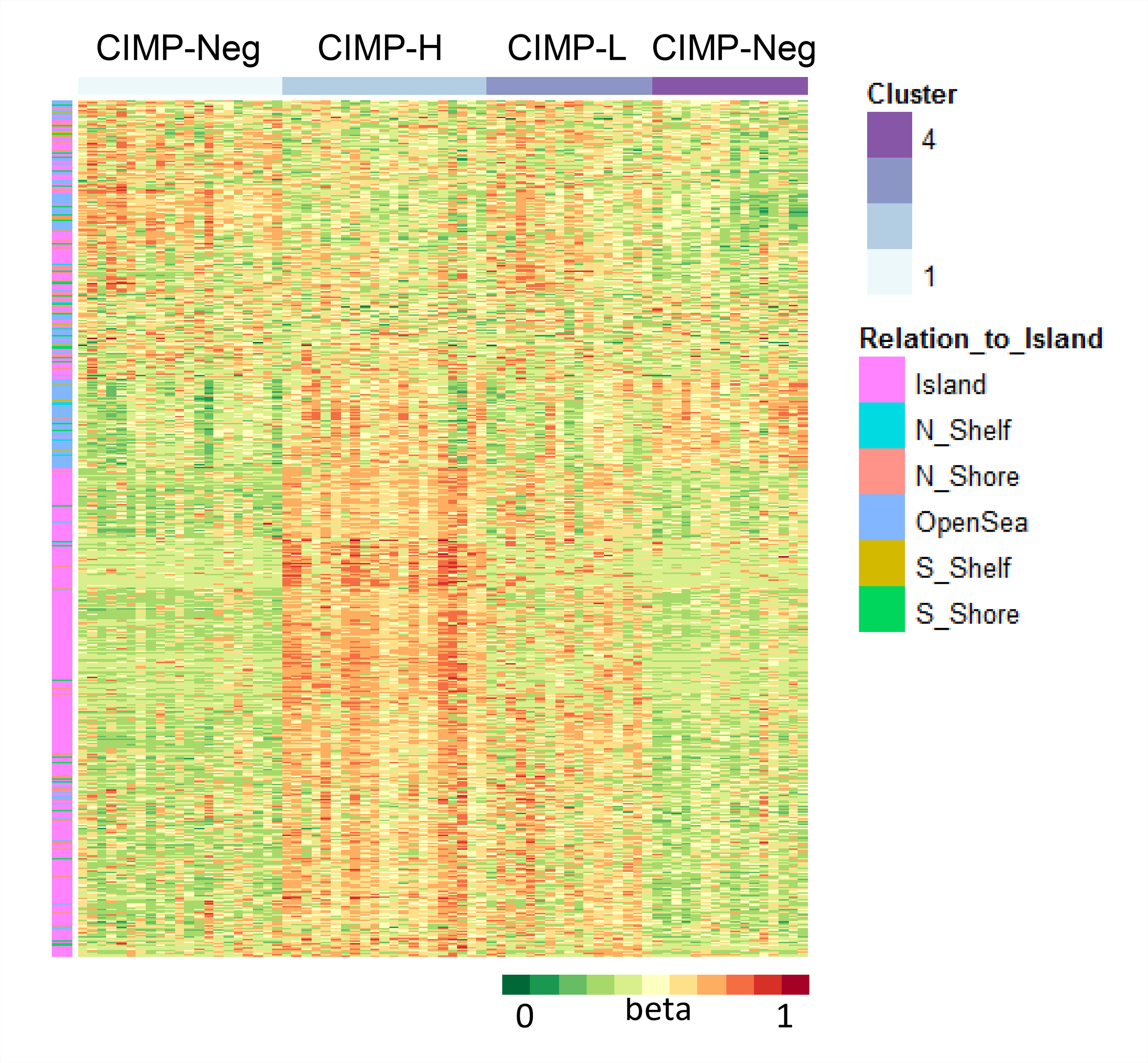

Supplement: FIGURE S2 [file OncolRes-31-30374-s002.tif]

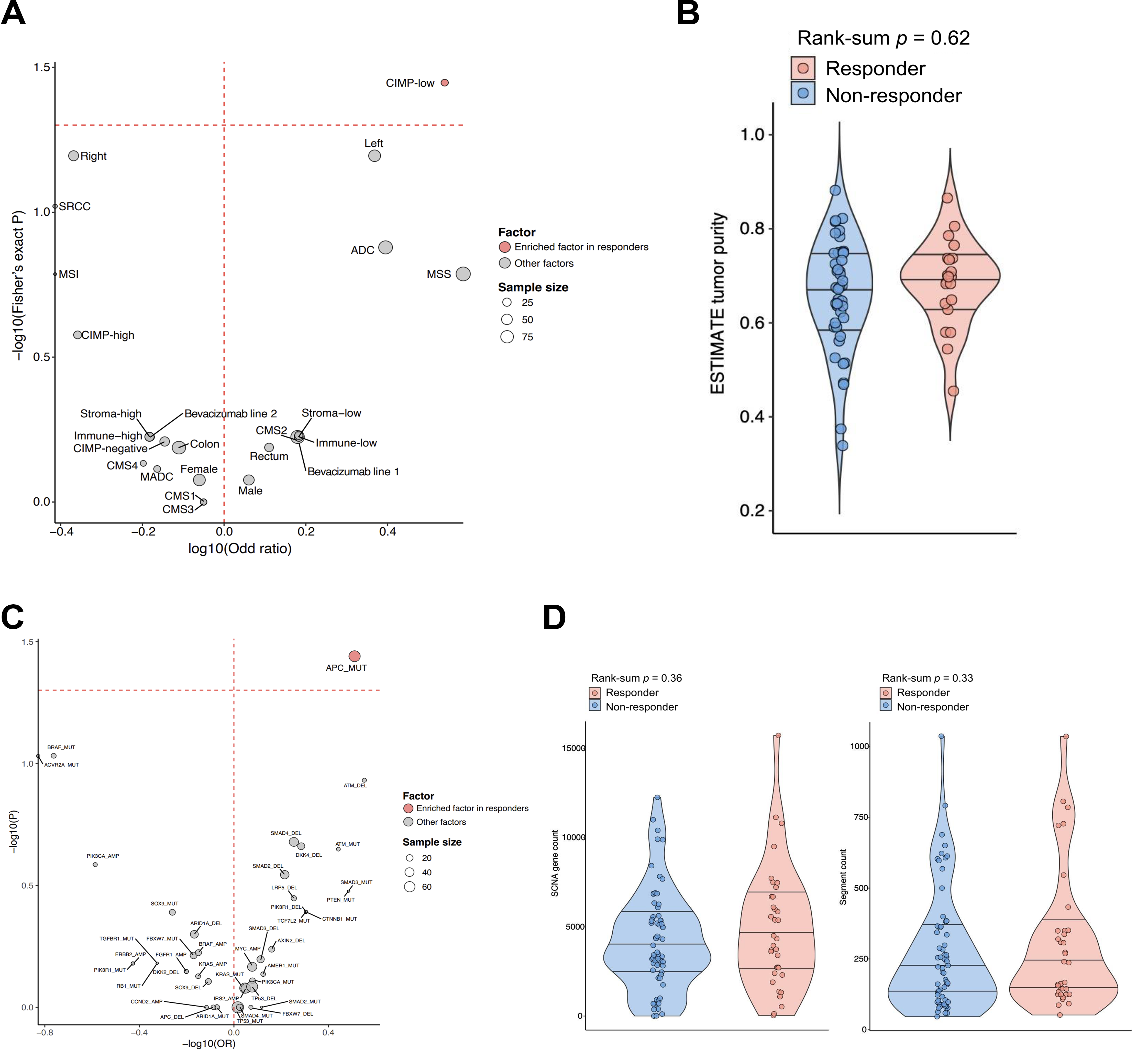

Supplement: FIGURE S3 [file OncolRes-31-30374-s003.tif]

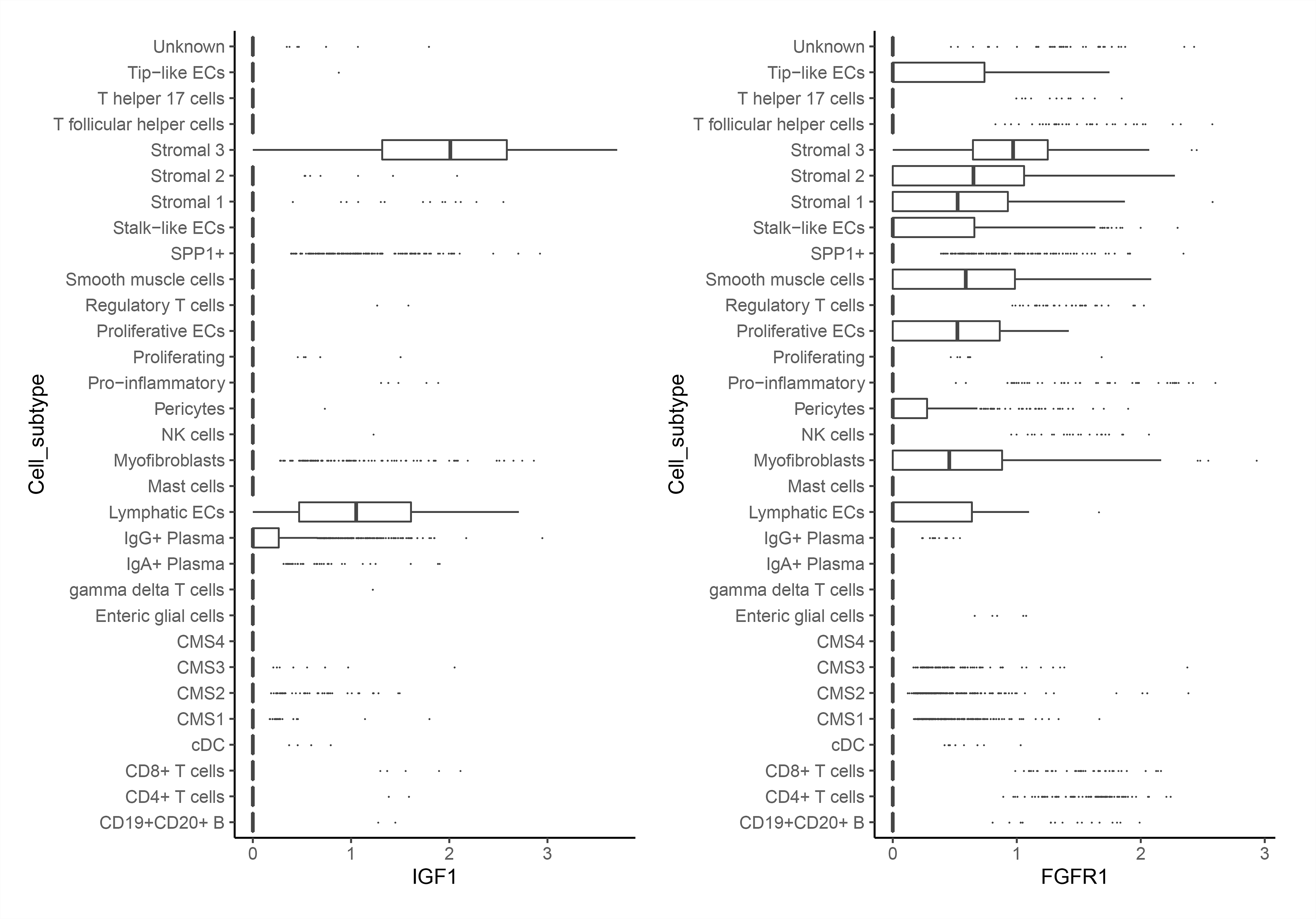

Supplement: FIGURE S4 [file OncolRes-31-30374-s004.tif]

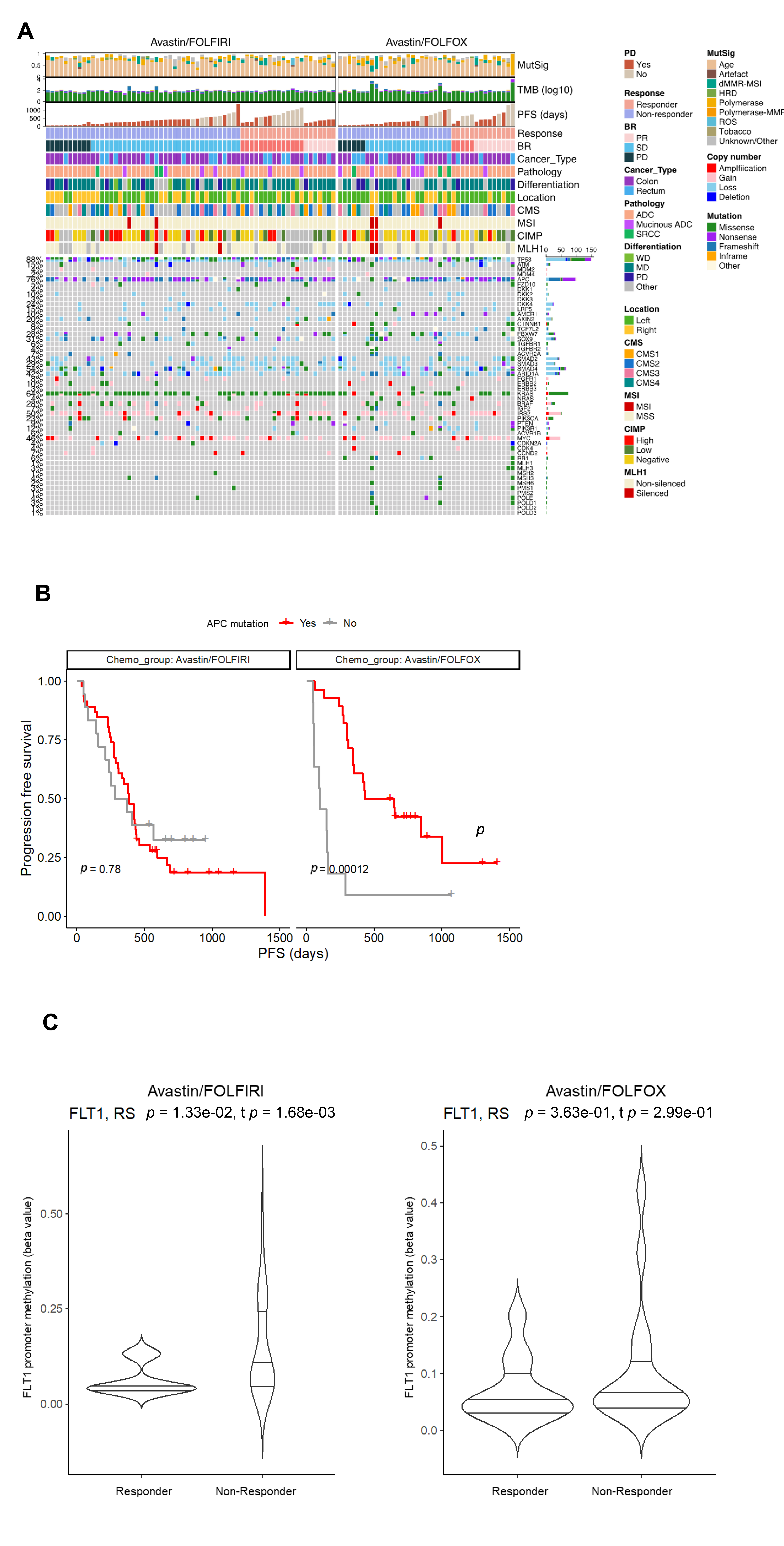

Supplement: FIGURE S5 [file OncolRes-31-30374-s005.tif]

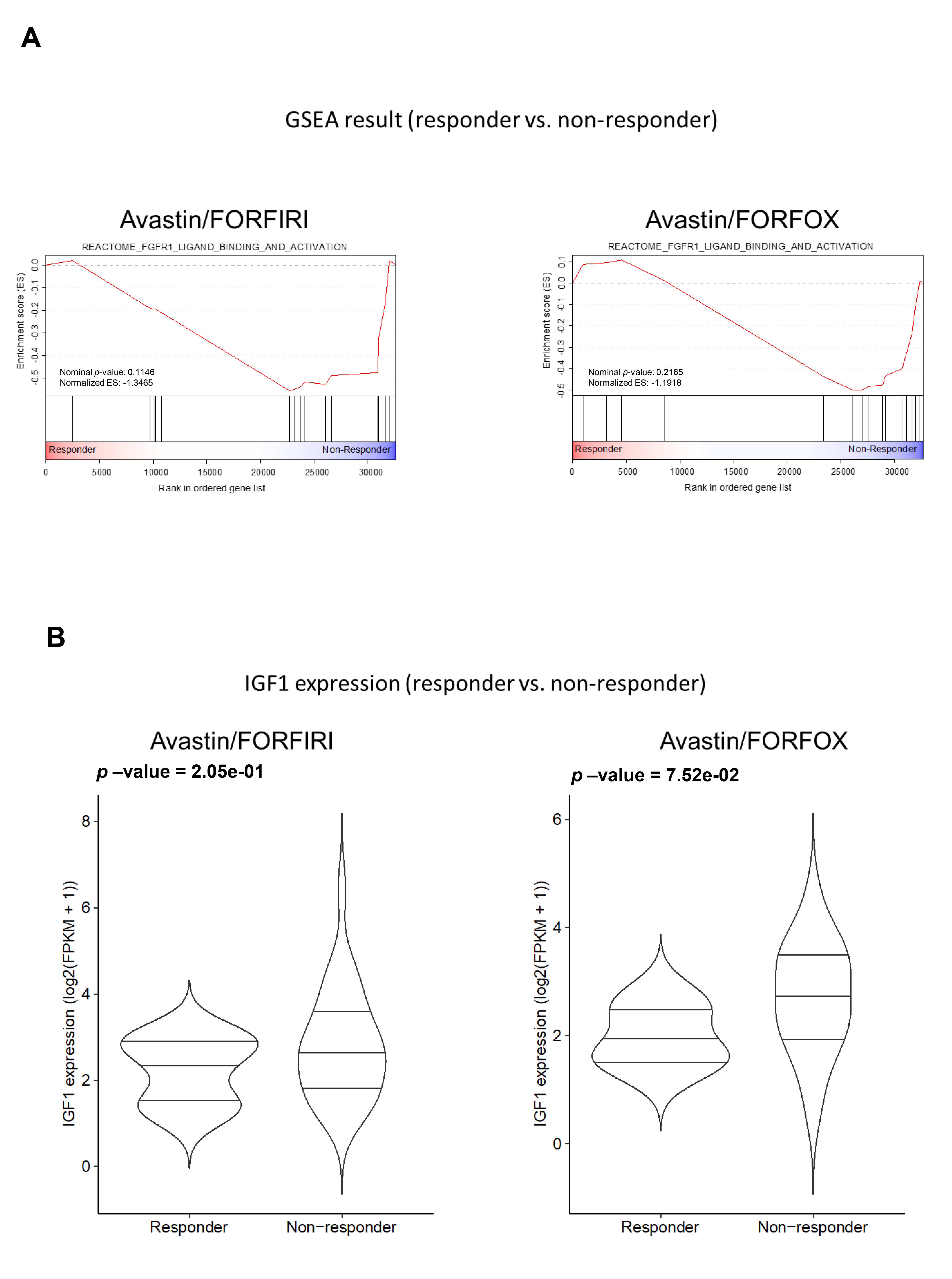

Supplement: FIGURE S6 [file OncolRes-31-30374-s006.tif]
